# Non-invasive PD-L1 stratification in non-small cell lung cancer using dynamic contrast-enhanced MRI

**DOI:** 10.1007/s00330-025-11524-1

**Published:** 2025-03-27

**Authors:** Gaia Messana, Chandra Bortolotto, Sithin Thulasi Seetha, Alessandra Marrocco, Carlotta Pairazzi, Francesco Sanvito, Francesca Brero, Agnese Robustelli Test, Raffaella Fiamma Cabini, Alessandro Lascialfari, Domenico Zacà, Giulia Maria Stella, Francesco Agustoni, Jessica Saddi, Andrea Riccardo Filippi, Lorenzo Preda

**Affiliations:** 1 Diagnostic Imaging and Radiotherapy Unit, Department of Clinical, Surgical, Diagnostic, and Pediatric Sciences, University of Pavia, Pavia, Italy; 2 Radiology Institute, Fondazione IRCCS Policlinico San Matteo, Pavia, Italy; 3 Clinical Department, National Center for Oncological Hadrontherapy (CNAO), Pavia, Italy; 4 UCLA Brain Tumor Imaging Laboratory (BTIL), Center for Computer Vision and Imaging Biomarkers, University of California Los Angeles, Los Angeles, CA USA; 5 Department of Radiological Sciences, David Geffen School of Medicine, University of California Los Angeles, Los Angeles, CA USA; 6 Department of Physics, University of Pavia, Pavia, Italy; 7 Istituto Nazionale Di Fisica Nucleare, Sezione Di Pavia, Pavia, Italy; 8 Euler Institute, Università della Svizzera Italiana, Lugano, Switzerland; 9 Siemens Healthcare srl, Milano, Italy; 10 Department of Medical Sciences and Infective Diseases, Unit of Respiratory Diseases, Fondazione IRCCS Policlinico San Matteo, Pavia, Italy; 11 Department of Internal Medicine and Medical Therapeutics, University of Pavia, Pavia, Italy; 12 Department of Medical Oncology, Fondazione IRCCS Policlinico San Matteo, Pavia, Italy; 13 Radiation Oncology, Fondazione IRCCS Policlinico San Matteo, Pavia, Italy; 14 Radiation Oncology, Fondazione IRCCS Istituto Nazionale dei Tumori, Milan, Italy; 15 Department of Oncology, University of Milan, Milan, Italy

**Keywords:** Non-small cell lung carcinoma, Immunotherapy, PD-L1 inhibitors, Perfusion magnetic resonance imaging, Pharmacokinetics

## Abstract

**Objectives:**

This study aimed to assess whether pharmacokinetic parameters derived from DCE-MRI can stratify Programmed Death-Ligand 1 (PD-L1) expression in NSCLC. The secondary aim was to identify a suitable pharmacokinetic model configuration for anisotropic temporally-spaced DCE-MRI sequences, considering Tofts variants, population-averaged arterial input functions (AIF), and bolus arrival time (BAT) estimation methods.

**Materials and methods:**

From April 2021 to May 2023, patients with locally advanced non-small cell lung cancer (NSCLC) were prospectively enrolled. Tumors were categorized based on: PD-L1 absence/presence (threshold 1%) and hyperexpression/hypoexpression (threshold 50%). Pharmacokinetic parameters were extracted using several candidate configurations; fit quality was evaluated using coefficient of determination (*R*²). Mann–Whitney U-test and ROC-AUC were used to assess correlation with PD-L1 for the best-fit configuration.

**Results:**

Thirty-eight patients (mean age 68 ± 9 years, 28 men) were included. PD-L1 expression was present in 25 patients (66%) and absent in 13 (34%). PD-L1 was hyperexpressed in 13 (34%) patients and hypoexpressed in 25 (66%). Voxel-wise pharmacokinetic parameters were extracted using the best-fit configuration—extended Tofts model (ETM) with Georgiou AIF and Peak-Gradient (PG) BAT estimation (*R*^2^ = 0.79). K^trans^ median (0.25 vs. 0.12 min^−^¹, *p* = 0.02), K^trans^ standard deviation (0.32 vs. 0.23 min^−^¹, *p* = 0.01) and K_ep_ median (1.09 vs. 0.59 min^−^¹, *p* = 0.02) were significantly higher in PD-L1 < 50% group (ROC-AUC 0.71–0.76).

**Conclusion:**

DCE-MRI pharmacokinetic parameters could stratify PD-L1 hypo/hyperexpression in NSCLC. The ETM with PG BAT estimation method and Georgiou AIF was the best-performing pharmacokinetic configuration.

**Key Points:**

***Question*** *Could Dynamic Contrast-Enhanced (DCE) MRI offer a safe and non-invasive way to assess Programmed Death-Ligand 1 (PD-L1) expression?*

***Findings*** *Quantitative DCE-MRI parameters K*^trans^ (the volume transfer rate) and K_ep_ (the efflux rate constant) show potential for distinguishing PD-L1 hyperexpression from hypoexpression.

***Clinical relevance*** *Preliminary results suggest that DCE-MRI could be a safe method to stratify PD-L1 hypo/hyperexpression in non-small cell lung cancer, potentially optimizing treatment decisions, given the high cost of immunotherapy.*

**Graphical Abstract:**

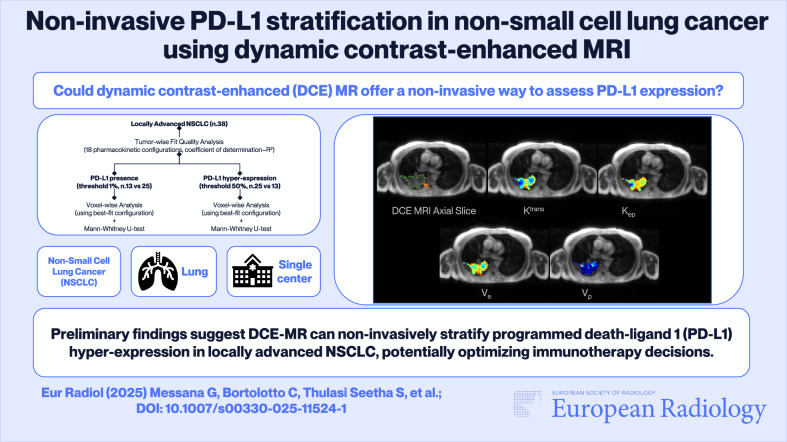

## Introduction

Lung cancer is the most prevalent cancer globally and the leading cause of cancer-related deaths worldwide [1]. Non-small cell lung cancer (NSCLC) accounts for nearly 85% of all lung cancer cases. In NSCLC, immune checkpoint inhibitors (ICIs) targeting programmed cell death protein 1 (PD-1) or its ligand (PD-L1) have shown greater efficacy and lower toxicity compared to chemotherapy [2–4]. However, only 20–50% of patients achieve long-term positive treatment responses [5, 6]. This highlights the importance of accurate patient stratification before administering ICIs, to optimize therapeutic outcomes. The National Comprehensive Cancer Network (NCCN) currently recommends stratifying patients with NSCLC for immunotherapy based on PD-L1 expression, determined through tumor proportion score (TPS) via immunohistochemical (IHC) analysis on pathological samples [7]. For example, the main indications for the treatment with Pembrolizumab, a PD-L1 inhibitor, are: (1) as a first-line therapy for metastatic NSCLC with PD-L1 TPS ≥ 50% and (2) treatment for patients with metastatic NSCLC whose tumors express PD-L1 (TPS ≥ 1%), with disease progression or after chemotherapy [8]. Besides, numerous clinical trials have demonstrated the predictive value of PD-L1 TPS for treatment response [4, 9].

However, bioptic procedures carry significant risks and may not represent the overall PD-L1 status of the tumor [10]. Therefore, non-invasive, safer, and repeatable alternative methods to assess PD-L1 expression are needed.

In recent years, Dynamic Contrast-Enhanced MRI (DCE-MRI) has gained significant interest due to its potential to extract biological characteristics of tumors. DCE-MRI provides information on tumor microvascular properties, such as perfusion and permeability, and it has been applied in many cancer types, including breast, head and neck, and gastric tumors [11–14].

Lung MRI has historically been challenging due to the intrinsic properties of the organ, such as low proton density and movement artifacts. However, recent advancements in MRI sequences, such as improved gradient performance and free-breathing acquisitions, have increased interest in lung MRI. To our knowledge, no studies have examined the correlation between DCE-MRI pharmacokinetic parameters and PD-L1 expression in NSCLC.

The primary aim of this study was to investigate whether DCE-MRI pharmacokinetic parameters can stratify PD-L1 expression in NSCLC. The secondary aim was to identify the best configuration of the pharmacokinetic model for the DCE-MRI sequences designed for fast acquisition with anisotropic temporal resolution.

## Materials and methods

### Patient data

The study was conducted following the Declaration of Helsinki and approved by the Institutional Ethics Committee of Fondazione IRCCS Policlinico San Matteo. Informed consent was obtained from all subjects involved in the study.

From April 2021 to May 2023, we prospectively enrolled a consecutive cohort comprising patients diagnosed with locally advanced NSCLC (according to TNM classification VIII edition, stage III A-C, and T ≥ 2). Exclusion criteria were: (1) insufficient compliance to ensure completion of the MR examination; (2) any treatment before undergoing MRI; (3) absence of PD-L1 expression test. PD-L1 expression was quantified through the 22C3 pharmDx assay (Agilent Technologies) [15] and TPS was evaluated according to guidelines [16]. Tumors were categorized based on—(1) the presence/absence of PD-L1 expression (TPS ≥ 1% and TPS < 1%) and (2) PD-L1 hyperexpression/hypoexpression (TPS ≥ 50% and TPS < 50%).

### MRI acquisition protocol

**Fig. 1:**
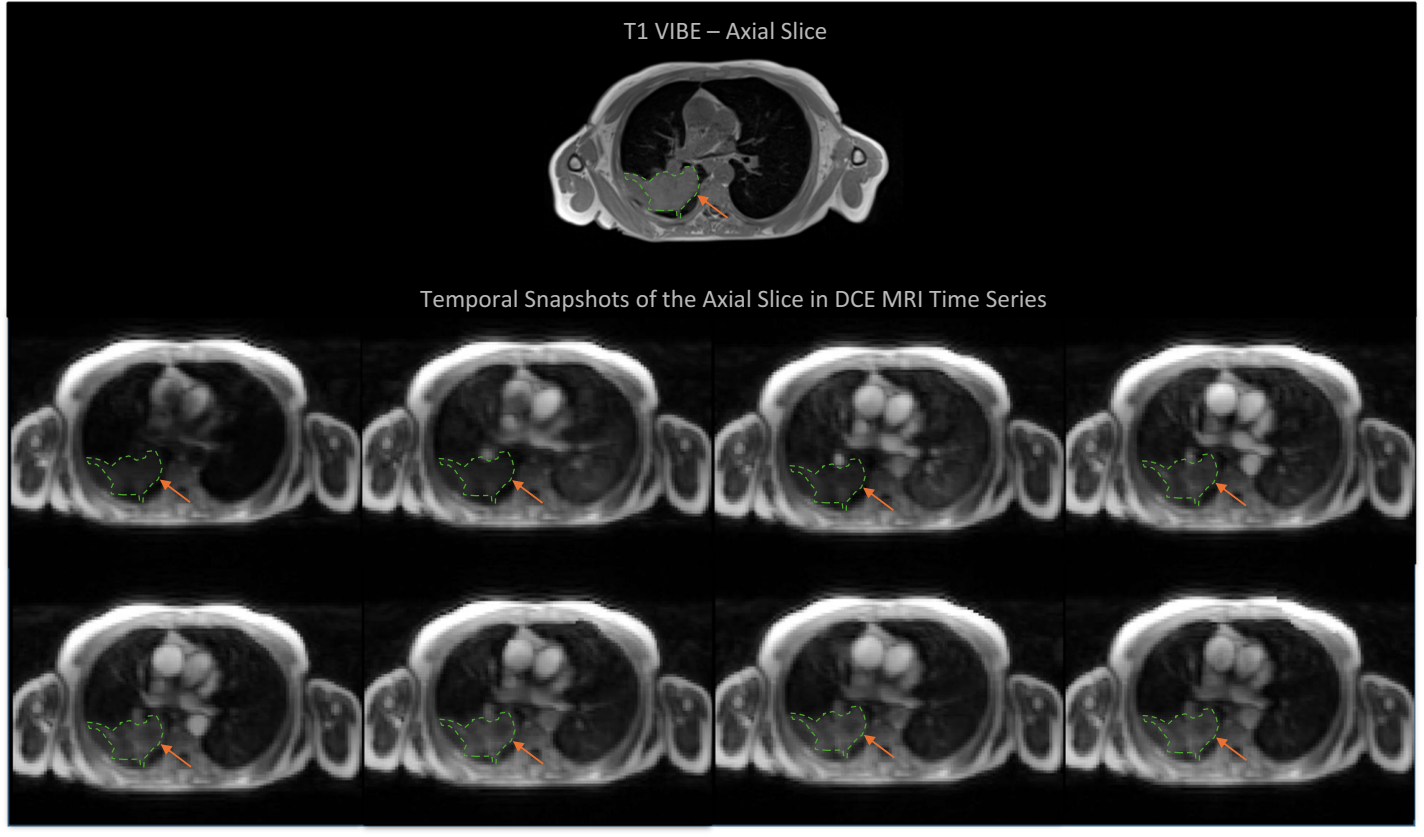
Eight out of 30 images illustrating dynamic vascular changes over time for a sample non-breath-hold TWIST DCE-MRI time series. Annotations highlight the tumor. T1 VIBE axial slice has been included as a reference to illustrate tumor morphology

MRI was performed using a 1.5-Tesla scanner (MAGNETOM Aera; Siemens Healthcare) using an 18-channel surface coil. Scan sequences included volumetric interpolated breath-hold examination (VIBE) T1 sequences and free-breathing DCE sequences. The DCE-MR acquisition (Table 1) was based on time-resolved angiography with interleaved stochastic trajectories (TWIST). A bolus of gadoteric meglumine (Dotarem), at a clinical standard dose of 0.2 mL/kg, was injected intravenously at a rate of 3–4 mL/s (Fig. 1).

**Table 1:** Main features of dynamic contrast-enhanced MRI (DCE-MRI) sequence

DCE-MRI acquisition parameters	
Feature	Value [median; interquartile range]
Matrix	128 × 128
Repetition time (TR)	2.24–2.27 ms [2.25; 0.01]
Time to echo (TE)	0.73–0.74 ms [0.74; 0.01]
Field of view (FOV)	384–400 mm [400; 0.0]
Slice spacing	3 mm
Pixel-spacing	3.00–3.12 mm [3.12; 0.0]
Number of layers	30
Temporal resolution	1.33–84.23 s [1.34; 0.0]
Flip angle	23–25° [25; 0.0]
Number of measurements	30
Duration	0.8–3.48 min [1.47; 0.07]

### Image preprocessing and segmentation

To reduce motion errors due to respiration and cardiac movement, we employed SimpleITK’s diffeomorphic demons registration filter [17]. Tumor delineation was performed semi-automatically. Initially, an experienced radiologist segmented the tumor on pre-contrast VIBE images using ITK-SNAP software (http://www.itksnap.org). The segmentation mask was then aligned with the DCE-MRI series using SimpleITK’s non-rigid BSpline registration technique, and any errors were manually corrected.

### Arterial input functions

We explored three popular population arterial input functions (AIFs) proposed by Weinmann, Parker, and Georgiou. Each has a parameterized functional form, formulated by aggregating subject-wise AIFs extracted from DCE-MRI acquired with high temporal resolution [18–20] (Supplementary material (SM), section S1).

### Bolus arrival time estimation

**Fig. 2:**
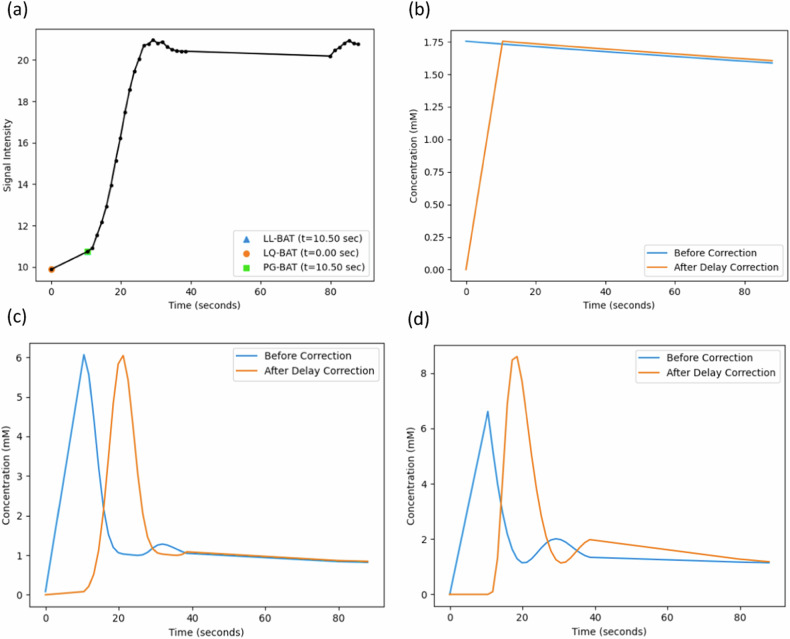
**a** Comparison of the three different bolus arrival time (BAT) estimation methods—LinearLinear (LL), LinearQuadratic (LQ) and PeakGradient (PG) on the mean tumor signal for a sample patient; Subsequent visualization of delay corrected population arterial input functions—**b** Weinmann, **c** Parker, and **d** Georgiou. For visualizing delay correction, we used the BAT estimated using the PG method

Bolus arrival time (BAT, t_0_) estimation was incorporated into the pharmacokinetic model to enhance the fit. To ensure stability, BAT was initialized using automated BAT estimation methods applied to the tumor-derived mean time-signal intensity curve (TIC). Three commonly used methods were evaluated: Linear-Linear (LL), Linear-Quadratic (LQ), and Peak-Gradient (PG) (Fig. 2) (SM, section S2).

### Pharmacokinetic modeling

We investigated two widely used variations of the Tofts bicompartmental model—the standard Tofts model (TM) and extended Tofts model (ETM)—to characterize tumor microvascular properties. Pharmacokinetic parameters, including K^trans^ (the volume transfer rate), K_ep_ (the efflux rate constant), V_e_ (the extracellular extravascular fraction), and V_p_ (the blood plasma volume fraction, only present in ETM), were optimized to fit the time-concentration curves (TCCs) using the Trust Region Reflective algorithm [21]. The steps involved in voxel-wise (tumor-wise) pharmacokinetic modeling can be summarized as follows: (1) Convert voxel-wise (mean-tumor) TIC to voxel-wise (mean-tumor) TCC; (2) Optimize the model parameters to fit the measured voxel-wise (mean-tumor) TCC; (3) For voxel-wise analysis, further compute the median, 90th percentile and standard deviation (SD) of non-necrotic voxels present within the target lesion; (4) Perform statistical (fit quality) analysis (SM, section S3).

### Fit quality analysis

To identify the best pharmacokinetic modeling approach for temporally anisotropic sequences, we investigated popular choices in three different levels of Tofts model design using mean-tumor TIC: (1) Tofts variants—standard or extended, (2) input population AIF—Weinmann, Parker, or Georgiou, and (3) BAT estimation methods—LL, LQ, or PG, collectively constituting 18 different configurations.

To quantify fit quality, we used the coefficient of determination, *R*^2^. By excluding voxels with *R*^2^ ≤ 0, we eliminated the impact of necrotic or outlier (poor fit) voxels within the tumor.

### Inter-reader agreement

First-order pharmacokinetic parameters extracted from tumor voxels may be subjected to variations due to changes in tumor delineation. To address this, we conducted an inter-reader agreement analysis on a random sample of ten patients. A second experienced radiologist independently performed manual segmentations on DCE-MR images. The agreement was assessed using the concordance correlation coefficient (CCC), with features having CCC > 0.85 classified as highly reproducible.

### Statistical analysis

We performed a Kruskal-Wallis H-test to assess whether the extracted first-order pharmacokinetic features differ significantly between subgroups of tumor histologies. Mann–Whitney U-test was performed to check for significance in differences between the groups of PD-L1 absence/presence (PD-L1 thresholded at 1%) and hyperexpression/hypoexpression (PD-L1 thresholded at 50%). A *p*-value < 0.05 was considered statistically significant. The receiver operating characteristics area under the curve (ROC-AUC) was estimated to assess the discriminative performance. Python (v3.7.5) and SciPy packages were used for all statistical analysis. The Python-based implementation is provided as open-source and is available at https://github.com/sithin-unipv/lungmr_pk.git (accessed on 25/09/24).

### Confounding factors analysis

To evaluate the potential impact of histology and nodal status as confounders on the statistically significant pharmacokinetic parameters, we conducted a bivariable analysis using logistic regression, adjusted for nodal status and histology.

## Results

A total of 38 patients were included. Among them, 28 (74%) were males and 10 (26%) were females. All the patients were of Caucasian ethnicity, with an average age of 68 ± 9 years. There were 14 patients (36%) with lung adenocarcinoma, 12 (32%) with lung squamocellular carcinoma, and 12 patients (32%) with poorly differentiated NSCLC. Of these, 25 (66%) had a PD-L1 expression (≥ 1%), and 13 (34%) showed PD-L1 hyperexpression (≥ 50%). Table 2 summarizes the main patient characteristics.

**Table 2:** Summary of patients’ characteristics

Main patient characteristics (*n* = 38)	
Characteristic	Value
Age (years)	
Median	68
Range	50–85
Sex	
Female	26% (10)
Male	74% (28)
Histology	
Adenocarcinoma	36% (14)
Squamous cell carcinoma	32% (12)
NSCLC poorly differentiated	32% (12)
Nodal Status	
N0 (negative)	11% (4)
N1-N3 (positive)	89% (34)
PD-L1 expression	
PD-L1 ≥ 1%	66% (25)
PD-L1 < 1%	34% (13)
PD-L1 hyperexpression/hypoexpression	
PD-L1 ≥ 50%	34% (13)
PD-L1 < 50%	66% (25)

Pharmacokinetic parameters—K^trans^, K_ep_, V_e_, and V_p_ (only for ETM) were estimated by fitting 18 distinct model configurations on the mean-tumor TIC. The median *R*^2^ values ranged from 0.06 to 0.79 (Table 3). The ETM pharmacokinetic model with Georgiou AIF and PG BAT estimation method was the best-performing configuration, resulting in a median *R*^2^ of 0.79. This configuration was later used to extract ETM parametric maps.

**Table 3:** Results of the fit quality analysis for the 18 different configurations examined on the mean tumor contrast agent concentration curve

PK model	AIF	BAT	Median *R*^2^
Extended Tofts	Georgiou	LL	0.694
		LQ	0.653
		PG	0.789
	Parker	LL	0.702
		LQ	0.682
		PG	0.788
	Weinmann	LL	0.546
		LQ	0.454
		PG	0.527
Standard Tofts	Georgiou	LL	0.709
		LQ	0.660
		PG	0.765
	Parker	LL	0.679
		LQ	0.671
		PG	0.682
	Weinmann	LL	0.221
		LQ	0.303
		PG	0.065

*PK* pharmacokinetic, *AIF* arterial input function, *BAT* bolus arrival time, *R*^2^ coefficient of determination, *LL* linear-linear, *LQ* linear-quadratic, *PG* peak-gradient

Kruskal-Wallis H-test showed no statistically significant differences (*p* > 0.05) among first-order pharmacokinetic features derived from the parametric maps across tumor histologies (Table S1). Inter-reader agreement analysis revealed that the features median (K^trans^), median (K_ep_), median (V_e_), 90th percentile (V_e_), and SD (V_e_) were highly reproducible (CCC: 0.90–0.96) (Table S2).

For the 50% threshold of PD-L1 expression, there were significant differences in features extracted from the K^trans^ and K_ep_ maps. For K^trans^, higher values were observed in the hypoexpression PD-L1 group for the features: median (0.25 vs. 0.12 min^−^^1^, *p* = 0.02), 90th percentile (0.74 vs. 0.44 min^−^^1^, *p* = 0.01), and SD (0.32 vs. 0.23 min^−^^1^, *p* = 0.01). Similarly, for K_ep_, higher values were observed in the hypoexpression PD-L1 group for the features: median (1.09 vs. 0.59 min^−^^1^, *p* = 0.02) and 90th percentile (2.21 vs. 1.87 min^−^^1^, *p* = 0.04) (Table 4).

**Table 4:** Results for the 50% threshold of PD-L1 expression

Feature	Median (PD-L1 < 50%)	Median (PD-L1 ≥ 50%)	ROC-AUC	95% CI	Threshold	Acc	Sensitivity/Specificity	*p*-value
Median (K_ep_)	**1.089879**	**0.590350**	**0.729**	**[0.56, 0.88]**	**0.95**	**0.68**	**0.64/0.85**	**0.022791**
90th percentile (K_ep_)	**2.213419**	**1.875468**	**0.711**	**[0.53, 0.86]**	**2.78**	**0.63**	**0.48/0.92**	**0.036411**
Standard deviation (K_ep_)	1.347053	1.147729	0.594	[0.41, 0.78]	1.30	0.63	0.60/0.77	0.355967
Median (K^trans^)	**0.246861**	**0.120352**	**0.735**	**[0.56, 0.89]**	**0.18**	**0.68**	**0.68/0.77**	**0.019363**
90th percentile (K^trans^)	**0.740485**	**0.436491**	**0.726**	**[0.55, 0.88]**	**0.55**	**0.74**	**0.76/0.69**	**0.024694**
Standard deviation (K^trans^)	**0.321472**	**0.232454**	**0.757**	**[0.58, 0.90]**	**0.30**	**0.68**	**0.68/0.77**	**0.010654**
Median (V_e_)	0.243500	0.215956	0.585	[0.40, 0.76]	0.28	0.53	0.36/0.92	0.406104
90th percentile (V_e_)	0.414260	0.398898	0.572	[0.38, 0.75]	0.53	0.50	0.24/1.00	0.479136
Standard deviation (V_e_)	0.127310	0.119866	0.535	[0.34, 0.73]	0.12	0.58	0.64/0.54	0.735015
Median (V_p_)	0.008537	0.008100	0.526	[0.33, 0.73]	0.00	0.66	0.80/0.38	0.805563
90th percentile (V_p_)	0.049675	0.032970	0.609	[0.41, 0.79]	0.05	0.58	0.52/0.77	0.281515
Standard deviation (V_p_)	0.032906	0.024364	0.612	[0.41, 0.80]	0.03	0.58	0.72/0.62	0.267995

*PD-L1* programmed death ligand 1, *ROC-AUC* area under the receiver operating characteristic curve, *95% CI* 95% confidence interval of ROC-AUC, *Acc* accuracy

Statistically significant PK parameters’ rows (*p* < 0.05) are highlighted

**Fig. 3:**
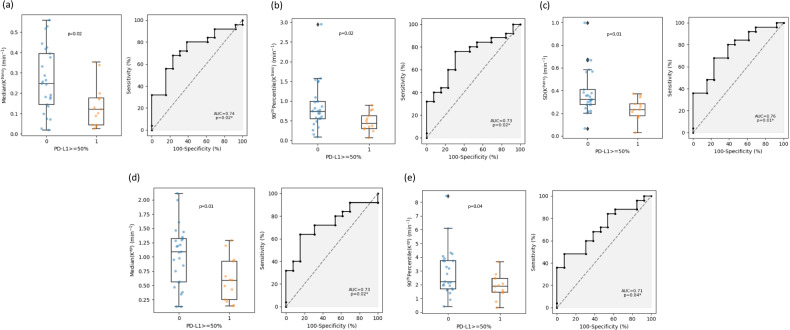
The distribution plot and ROC curves of statistically significant pharmacokinetic parameters identified in this study: **a** Median (K^trans^); **b** 90th percentile (K^trans^); **c** Standard deviation, SD (K^trans^); **d** Median (K_ep_), and **e** 90th percentile (K_ep_)

**Fig. 4:**
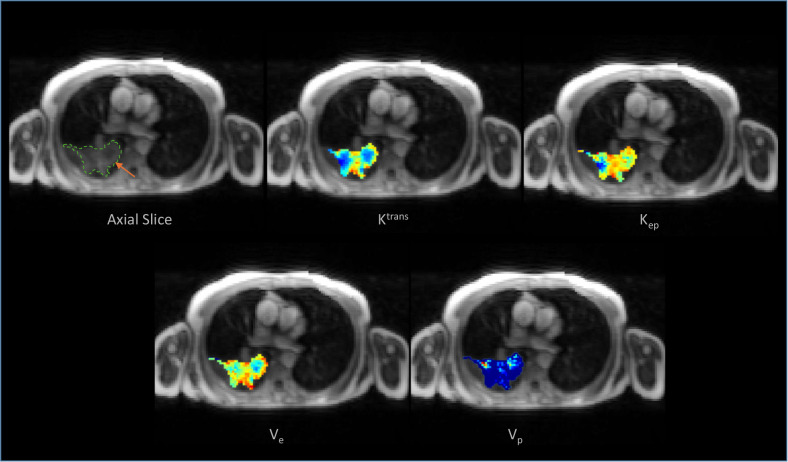
Illustration of a tumor-focalized DCE-MR axial slice selected from an example patient, along with the corresponding extended Tofts parametric maps extracted using Georgiou arterial input function and peak gradient bolus arrival time estimation method

Mann–Whitney U-tests for the 1% threshold revealed no statistical significance (*p* > 0.05) (Table 5). Figure 3 illustrates the distribution and ROC-AUC of the significant features identified in this study. Figure 4 presents the parametric maps extracted for a sample lesion. Of note, the ETM parameters estimated using the mean tumor TIC were close to the median parameter map estimates (Table S3).

**Table 5:** Results for the 1% threshold of PD-L1 expression

Feature	Median (PD-L1 < 1%)	Median (PD-L1 ≥ 1%)	ROC-AUC	*p*-value
Median (K_ep_)	0.973981	0.849796	0.507	0.950930
90th percentile (K_ep_)	2.007770	2.132588	0.545	0.666636
Standard deviation (K_ep_)	1.224932	1.240920	0.483	0.877731
Median (K^trans^)	0.246861	0.175889	0.523	0.829467
90th percentile (K^trans^)	0.693362	0.605147	0.535	0.735015
Standard deviation (K^trans^)	0.302964	0.298183	0.569	0.498455
Median (V_e_)	0.232556	0.238432	0.489	0.926454
90th percentile (V_e_)	0.413462	0.411794	0.501	1.000000
Standard deviation (V_e_)	0.120894	0.123767	0.486	0.902046
Median (V_p_)	0.008042	0.008537	0.529	0.781839
90th percentile (V_p_)	0.033862	0.045000	0.502	1.000000
Standard deviation (V_p_)	0.027926	0.036257	0.489	0.926454

*PD-L1* programmed death ligand 1, *ROC-AUC* area under the receiver operating characteristic curve

The bivariable logistic regression analysis on clinically significant features revealed that median(K_ep_), median(K^trans^), and SD(K^trans^) are independent predictors (*p* < 0.05), unaffected by histology or nodal status (Table S4).

## Discussion

In this study, we utilized short-period temporally anisotropic lung DCE-MRI sequences of NSCLC patients to investigate the role of pharmacokinetic parameters in the stratification of PD-L1 expression. Furthermore, we identified a suitable pharmacokinetic configuration for this custom sequence, in terms of choices in Tofts model type, BAT estimation method, and population AIF. We categorized patients according to two PD-L1 TPS thresholds: (1) PD-L1 ≥ 1%, reflecting the clinical eligibility criteria for immunotherapy, and (2) PD-L1 ≥ 50%, commonly used to identify patients with high PD-L1 expression who are more likely to benefit from immunotherapy. Our results demonstrated a negative correlation between K^trans^ and K_ep_ values with PD-L1 expression and showed moderate accuracy in discriminating PD-L1 hyperexpression. ETM with PG BAT estimation method and Georgiou AIF was the best-performing pharmacokinetic configuration.

DCE-MRI may be leveraged to quantify perfusion and microvascular parameters by fitting pharmacokinetic models to contrast concentration curves [22]. The principal quantitative parameters obtained from ETM are: K^trans^, the volume transfer constant of contrast agent leaked into extravascular extracellular space (EES) from plasma; K_ep_, the rate constant of contrast agent reflux to the plasma; V_e_, the EES volume fraction; and V_p_, the plasma volume fraction [23]. As vascular abnormality is one of the primary features of malignant tumors, DCE-MRI has been extensively evaluated to extrapolate the pharmacokinetic parameters associated with these diseases [24]. Nonetheless, there is a relatively low number of publications on the application of DCE-MRI to lung tumors due to its limited use in clinical practice and the technical challenges of lung MRI.

In the study of Wang et al elevated K^trans^ values correlated positively with better treatment response leading to extended progression-free survival (PFS) and overall survival (OS) in unresectable stage III NSCLC patients who underwent immunotherapy [25]; Sridharan et al showed that K^trans^ is repeatable and detects early treatment-induced changes in NSCLC lesions, supporting its use in the evaluation of biological response to radiotherapy and targeted therapies [26]. Regarding histological differentiation, Guo et al identified K^trans^ as a promising parameter to differentiate small-cell lung cancer from NSCLC and adenocarcinoma from squamous cell carcinoma [27]. Zou et al demonstrated that some K^trans^ and K_ep_ features were reliable in evaluating the epidermal growth factor receptor (EGFR) gene mutation status and the expression of vascular endothelial growth factor (VEGF) and EGFR proteins in NSCLC [28]. No previous studies have explored the correlation between DCE-MRI parameters and PD-L1 expression in lung cancer.

Our results showed significantly higher K_ep_ and K^trans^ values in patients with PD-L1 TPS < 50% compared to the group with PD-L1 TPS ≥ 50%, with moderate diagnostic accuracy, while being independent of confounding factors like histology and nodal status. NSCLC, like many other tumors, is characterized by the upregulation of pro-angiogenic factors. This results in the formation of an aberrant vascular network with increased vessel permeability, due to defective endothelial cell junctions and the overproduction of permeability-inducing agents such as VEGF [29, 30]. K^trans^ is a parameter that combines blood flow and permeability, and it has been shown to reflect blood flow under conditions of very high permeability, such as those found in tumors [31]; moreover, in this cohort of patients, the use of gadoteric acid, a lipophilic contrast agent that easily crosses the endothelial wall, further increases permeability [32]. K_ep_ is associated with microvessel density, and both K^trans^ and K_ep_ parameters can reflect tumor proliferation potential [33, 34]. Therefore, our results suggest that tumors with PD-L1 hyperexpression may have lower blood flow and reduced microvessel density compared to tumors with lower PD-L1 expression. This finding is in line with the study by Bortolotto et al [35], which reported an inverse correlation between D* values—representing the perfusion-related diffusion coefficient in intravoxel incoherent motion diffusion-weighted imaging (IVIM-DWI) and PD-L1 expression [35]. Also, our results are consistent with those of Franz et al who demonstrated a negative correlation between angiogenesis (marked by CD31) and PD-L1 expression in laryngeal carcinomas [36]. A possible explanation is that the greater infiltration of immune cells, likely present in tumors with higher PD-L1 expression [37], is associated with increased vessel permeability, leading to blood stasis and reduced blood flow in the local microcirculation. However, pharmacokinetic models provide results that are often difficult to interpret physiologically [32]. Of note, no statistically significant correlation for K_ep_ or K^trans^ had been found between groups for the PD-L1 TPS threshold of 1%.

PD-L1 expression status is necessary to determine whether a patient should receive immunotherapy as first- or second-line treatment. The 1% threshold establishes eligibility for ICIs, while the 50% threshold identifies patients who benefit most from first-line monotherapy with ICIs targeting the PD-1/PD-L1 axis. For PD-L1 < 50%, combination therapies with ICIs and chemotherapy are preferred to achieve faster responses. This makes the 50% threshold particularly relevant as it provides patient stratification for tailored treatment.

We performed inter-reader agreement analysis to assess the reproducibility of pharmacokinetic features to variations in segmentation. Semi-automatic and manual approaches allowed for greater variability in tumor delineation. Using a high CCC threshold (CCC > 0.85), we conservatively identified median K^trans^ and K_ep_ measures as highly reproducible, further supported by consistent results from mean tumor-wise analysis, reinforcing their reliability as robust imaging biomarkers.

The TWIST sequence accelerates DCE-MRl protocols which are better tolerated by patients with breathing difficulties, such as patients with lung cancer. Our secondary endpoint was to determine a suitable Tofts model configuration for this specific sequence, in terms of model type, AIF, and BAT estimation method. Bicompartmental pharmacokinetic models describe the contrast agent dynamics as the interaction between two compartments—vascular space and EES. The key difference between TM and ETM is that the latter extends the standard Tofts model by including V_p_ in the fit equation, which is particularly relevant in tissues like the lung parenchyma, where the high density of blood vessels can influence contrast agent dynamics.

The AIF, which refers to the concentration of the contrast medium in an artery over time, is a critical input for pharmacokinetic models [38] and can be subject-specific or population-based. We tested population-based AIF because the acquisitions were tumor-focalized, making subject-specific evaluation not feasible. Furthermore, the TWIST protocol, being anisotropic in time, reduces the accuracy of the estimation of subject-specific AIF, as it requires fixed high temporal resolution.

BAT represents the time taken for the contrast medium to reach the target vessel after intravenous injection [39]. Ignoring this parameter can impair pharmacokinetic fit quality [40]. While the Quantitative Imaging Biomarkers Alliance (QIBA) recommends correcting for this delay in the AIF, estimating BAT can be challenging in noise-prone short-duration anisotropically spaced TICs [41]. Hence, we included the delay as an additional parameter in the pharmacokinetic model.

Our analysis revealed that the ETM using either the Georgiou or Parker AIF with PG BAT estimation may be better suited for temporally anisotropic TWIST protocols (*R*^2^ ~ 0.79). Further studies may benefit from these results, integrating them with concurrent literature, to ease the analysis pipeline for DCE sequences in the lung cancer setting.

This study, given its preliminary and exploratory nature, has several limitations. First, it is a monocentric study with a relatively small sample size. As a result, the findings may not be fully representative of a larger population and require external validation with data obtained from other centers to ensure their generalizability and reliability. Second, the low availability of lung MRI limits its accessibility for widespread clinical use. However, DCE may be a promising sequence for evaluating the intrinsic properties of tumors, also in association with other MRI sequences. MRI can be repeated regularly, even during therapy, without the use of ionizing radiation; furthermore, this DCE-MRI protocol can be performed in short scanning times, thus it can be easily incorporated into MR exams. Third, the conservative inter-reader agreement analysis is simply indicative but can yield false negatives and requires rigorous validation on a larger population. Lastly, the diagnostic accuracy of K_ep_ and K^trans^ values in distinguishing between PD-L1 hypoexpression and hyperexpression was moderate, suggesting their potential utility, though not definitive. Future studies should aim to include larger, multicentric cohorts with standardized protocols to confirm these findings.

## Conclusions

Quantitative DCE-MRI parameters are potential imaging biomarkers for non-invasively stratifying PD-L1 hyperexpression in NSCLC. We highlight a methodology for choosing among different pharmacokinetic model combinations and identify the most appropriate configuration for temporally anisotropic DCE-MRI sequences.

## Supplementary information

ELECTRONIC SUPPLEMENTARY MATERIAL

## Abbreviations

AIF: Arterial input function
BAT: Bolus arrival time
CCC: Concordance correlation coefficient
DCE: Dynamic contrast-enhanced
DCE-MRI: Dynamic contrast-enhanced magnetic resonance imaging
EGFR: Epidermal growth factor receptor
ETM: Extended Tofts model
ICI: Immune checkpoint inhibitor
LL: Linear-linear
LQ: Linear-quadratic
NCCN: National Comprehensive Cancer Network
NSCLC: Non-small cell lung cancer
PD-L1: Programmed death-ligand 1
PG: Peak-gradient
QIBA: Quantitative Imaging Biomarkers Alliance
ROC-AUC: Receiver operating characteristics area under the curve
SD: Standard deviation
SM: Supplementary materials
TCC: Time-concentration curve
TIC: Time-signal intensity curve
TM: Standard Tofts model
TPS: Tumor proportion score
TWIST: Time-resolved angiography with interleaved stochastic trajectories
VEGF: Vascular endothelial growth factor
VIBE: Volumetric interpolated breath-hold examination
