# Supplementary material for: Non-invasive PD-L1 stratification in non-small cell lung cancer using dynamic contrast-enhanced MRI
Source: Eur Radiol. 2025 Mar 27;35(9):5569–80. doi: 10.1007/s00330-025-11524-1 (PMC12350491; doi:10.1007/s00330-025-11524-1)
Supplement: Supplementary file 1 — ELECTRONIC SUPPLEMENTARY MATERIAL [file 330_2025_11524_MOESM1_ESM.pdf]

Non-Invasive PD-L1 Stratification in Non-Small Cell Lung Cancer using Dynamic Contrast-Enhanced MRI
ELECTRONIC SUPPLEMENTARY MATERIAL

Section S1: Population AIFs

(a) Weinmann AIF

Input: Time Array, t in seconds; BAT, t_0

Step 1: Delay correction, $t = t - t_0$

Step 2: Convert t from seconds to minutes ($t = t/60$)

Step 3: Define constants: $A_1 = 3.99$; $A_2 = 4.78$; $M_1 = 0.144$; $M_2 = 0.0111$; $D = 0.2$

Step 4: Compute C_P using the formula: $C_P = D * [A_1 * \exp(-M_1 * t) + A_2 * \exp(-M_2 * t)]$

Step 5: For $t < 0$, Set C_P to 0 and return C_P

(b) Parker AIF

Input: Time Array, t in seconds; BAT, t_0

Step 1: Delay correction, $t = t - t_0$

Step 2: Convert t from seconds to minutes ($t = t/60$)

Step 3: Define constants: $A_1 = 0.809$; $A_2 = 0.330$; $T_1 = 0.17046$; $T_2 = 0.365$; $\sigma_1 = 0.0563$; $\sigma_2 = 0.132$; $\alpha = 1.050$; $\beta = 0.1685$; $s = 38.078$, $\tau = 0.483$

Step 4: Compute C_P using the formula

$$Cp(t) = A_1 \cdot \frac{1}{\sigma_1\sqrt{2\pi}} \cdot \exp\left(-\frac{(t-T_1)^2}{2\sigma_1^2}\right) + A_2 \cdot \frac{1}{\sigma_2\sqrt{2\pi}} \cdot \exp\left(-\frac{(t-T_2)^2}{2\sigma_2^2}\right) + \alpha \cdot \frac{\exp(-\beta t)}{1+\exp(-s(t-\tau))}$$

Step 5: Return C_P

(c) Georgiou AIF

Input: Time Array, t in seconds; BAT, t_0

Step 1: Delay correction, $t = t - t_0$

Step 2: Convert t from seconds to minutes ($t = t/60$)

Step 3: Define constants: $a_1 = 0.37$; $a_2 = 0.33$; $a_3 = 10.06$; $m_1 = 0.11$; $m_2 = 1.17$; $m_3 = 16.02$; $\alpha = 5.26$; $\beta = 0.032$; $\tau = 0.129$

Step 4: compute no. of circulations, n_{circ} = round_to_integer(t_{max}/τ)

Step 5: For each circulation, current_circ from 0 to $n_{circ}+1$, do steps 5.1 to 5.4

Step 5.1: Estimate timeindex = {arg max [(t >= current_circ *τ) & (t < (current_circ+1)*τ)], when current_circ < n_{circ}; otherwise arg max[(t >= current_circ * τ)]}

Step 5.2: Assign current_time = t[timeindex]

Step 5.3: Sum the contributions of the previous circulations, f_{total}

Step 5.4: Compute C_P[timeindex] as follows:

exp1 = a_1 * exp(-m_1 * current_time

exp2 = a_2 * exp(-m_2 * current_time)

exp3 = a_3 * exp(-m_3 * current_time)

sumexp = exp1 + exp2 + exp3

C_P[timeindex] = sumexp*f_{total}

Step 6: Return C_P

Section S2: BAT Estimation Methods

(a) Linear-Linear (LL)

Input: Time Array, t; T1w Signal Intensity (or Contrast Agent Concentration) Over Time, *S(t)*;

Step 1: Let [lower_bound, upper_bound] be the search internal* indices where t[lower_bound], t[upper_bound] $\in t$

Step 2: Let y = S[lower_bound: upper_bound], t = t[lower_bound: upper_bound]

Step 3: For each index, k from lower_bound to upper_bound, do steps 3.1 to 3.2

Step 3.1: Solve the equation $\hat{y}$ = {β_0 for t < t[k]; β_0+β_1*(t-t[k]) otherwise}

Step 3.2: Compute the error, SSE[k] between y and $\hat{y}$

Step 4: BAT = lower_bound + arg min (SSE)

(b) Linear-Quadratic (LQ)

Input: Time Array, t; T1w Signal Intensity (or Contrast Agent Concentration) Over Time, *S(t)*;

Step 1: Let [lower_bound, upper_bound] be the search internal* indices where t[lower_bound], t[upper_bound] $\in t$

Step 2: Let y = S[lower_bound: upper_bound], t = t[lower_bound: upper_bound]

Step 3: For each index, k from lower_bound to upper_bound, do steps 3.1 to 3.2

Step 3.1: Solve the equation $\hat{y}$ = {β_0 for t < t[k]; β_0+β_1*(t-t[k])+β_2*(t-t[k])^2 otherwise}

Step 3.2: Compute the error, SSE[k] between y and $\hat{y}$

Step 4: BAT = lower_bound + arg min (SSE)

(c) Peak-Gradient (PG)

Input: Time Array, t; T1w Signal Intensity (or Contrast Agent Concentration) Over Time, *S(t)*;

Step 1: Perform smoothing on the input Signal or Conc. data using Savizky-Golay filter

Step 2: Compute the spatial derivative of smoothed data independent of ascent/descent;

Step 3: Find the index of the point of steepest descent/ascent (extremum point)

Step 4: BAT = extremum point index ± tolerance

*vanilla search interval can be estimated as: lower_bound = argmin (S), upper_bound = argmax (S)

Section S3: Pharmacokinetic Modelling

Input: Time Array, t; T1w Signal Intensity Over Time, *T1w(t)*;
Repetition Time, TR; Flip Angle, FA;
Contrast Agent Relaxivity, r1; Assumed T1 relaxation time, T1(0);
Basepoint pre-contrast time indices–start, end

Step 1: Conversion of time-signal intensity curve to contrast agent concentration curve using the following equations:

Step 1.1: Pre-contrast T1w, T1w(0) = $\frac{1}{(end-start)}\sum_{t=start}^{end}$ T1w(t)

Step 1.2: Compute E1(0) = exp [-TR / T1(0)]

Step 1.3: B = [1 - E1(0)] / [1 - cos FA * E1(0)]

Step 1.4: Normalize by pre-contrast signal, A(t) = B * T1w(t) / T1w(0)

Step 1.5: fraction = [1 - A(t)] / [1 - cos FA * A(t)]

Step 1.6: R1(t) = TR^{-1} * ln (fraction)

Step 1.7: Baseline relaxation rate, R1(0) = 1 / T1(0)

Step 1.8: ΔR1(t) = R1(t) - R1(0)

Step 1.9: Contrast agent concentration over time, C_t(t) = ΔR1(t) / r1

Step 2: Optimize the pharmacokinetic parameters to fit the measured TCC to the following Tofts equations:

(a) Standard Tofts:

$$C_t(t) = K^{trans} \int_0^t C_p(\tau - t_0) \exp\left(-\frac{K^{trans}}{v_e}(t-\tau)\right) d\tau$$

Where,

- C_P (τ - t_0) is the delay-corrected AIF, where t_0 accounts for the delay
- K^{trans} is the transfer constant between plasma and extravascular-extracellular space (EES)
- V_e is the volume fraction of the EES
- $K_{ep} = K^{trans}/V_e$

(b) Extended Tofts:

$$C_t(t) = v_p C_p(t - t_0) + K^{trans} \int_0^t C_p(\tau - t_0) \exp\left(-\frac{K^{trans}}{v_e}(t-\tau)\right) d\tau$$

Where,

- V_p is the blood plasma volume fraction

Table S1: Summary statistics and Kruskal-Wallis H test results for PK features across histological types.

**Feature**	**Subtype=All (n.38) Median [IQR]**	**Subtype=ADK (n.14) Median [IQR]**	**Subtype=SCC (n.12) Median [IQR]**	**Subtype=NSCLC Poorly Diff. (n.12) Median [IQR]**	**P-values**
Median (K_{ep})	0.93 [0.47, 1.27]	1.19 [0.61, 1.31]	0.48 [0.24, 1.3]	0.93 [0.64, 1.08]	0.339053
90th Percentile (K_{ep})	2.1 [1.6, 3.56]	2.77 [1.71, 3.73]	1.97 [1.31, 2.64]	2.07 [1.68, 2.64]	0.536980
Standard Deviation (K_{ep})	1.23 [0.85, 1.56]	1.42 [1.25, 1.62]	1.19 [0.96, 1.35]	0.92 [0.67, 1.48]	0.331188
Median (K^{trans})	0.18 [0.1, 0.34]	0.24 [0.12, 0.37]	0.12 [0.04, 0.24]	0.19 [0.16, 0.3]	0.275164
90th Percentile (K^{trans})	0.62 [0.38, 0.85]	0.66 [0.5, 0.88]	0.4 [0.25, 1.01]	0.72 [0.54, 0.77]	0.497486
Standard Deviation (K^{trans})	0.3 [0.23, 0.37]	0.33 [0.22, 0.38]	0.28 [0.23, 0.37]	0.3 [0.25, 0.35]	0.935561
Median (V_e)	0.24 [0.2, 0.28]	0.24 [0.21, 0.28]	0.22 [0.2, 0.25]	0.26 [0.2, 0.28]	0.599613
90th Percentile (V_e)	0.41 [0.33, 0.48]	0.42 [0.35, 0.48]	0.37 [0.34, 0.4]	0.43 [0.33, 0.5]	0.384930
Standard Deviation (V_e)	0.12 [0.1, 0.14]	0.13 [0.09, 0.15]	0.12 [0.12, 0.13]	0.12 [0.1, 0.15]	0.992031
Median (V_p)	0.01 [0.0, 0.02]	0.01 [0.0, 0.02]	0.01 [0.0, 0.02]	0.01 [0.01, 0.04]	0.672851
90th Percentile (V_p)	0.04 [0.02, 0.09]	0.05 [0.02, 0.09]	0.04 [0.02, 0.08]	0.03 [0.02, 0.12]	0.926140
Standard Deviation (V_p)	0.03 [0.02, 0.06]	0.03 [0.02, 0.05]	0.03 [0.02, 0.06]	0.03 [0.02, 0.05]	0.982372

Table S2: Concordance correlation coefficient (CCC) of first-order features derived from ETM parametric maps obtained using the best-fit configuration.

Feature	CCC
Median (K_{ep})	0.967537
90th Percentile (K_{ep})	0.536256
Standard Deviation (K_{ep})	0.407855
Median (K^{trans})	0.963140
90th Percentile (K^{trans})	0.783031
Standard Deviation (K^{trans})	0.427014
Median (V_e)	0.946480
90th Percentile (V_e)	0.922098
Standard Deviation (V_e)	0.909051
Median (V_p)	0.263648
90th Percentile (V_p)	0.716411
Standard Deviation (V_p)	0.277551

Table S3: ETM parameters estimated by fitting the model to the mean tumor concentration curve: Statistical results for (a) PD-L1>=50%; (b) PD-L1>=1%

(a)

Feature	Median (PD-L1<50%)	Median (PD-L1≥50%)	p-value	ROC-AUC
**K_{ep}**	**1.099899**	**0.692627**	**0.019363**	**0.735385**
**K^{trans}**	**0.234481**	**0.111781**	**0.016395**	**0.741538**
V_e	0.232810	0.217762	0.441756	0.578462
V_p	0.012935	0.003469	0.666636	0.544615

(b)

Feature	Median (PD-L1<1%)	Median (PD-L1≥1%)	p-value	ROC-AUC
K_{ep}	1.096267	0.822861	0.926454	0.510769
K^{trans}	0.234481	0.167619	0.805563	0.526154
V_e	0.232810	0.220062	0.735015	0.535385
V_p	0.016226	0.004866	0.267995	0.612308

Table S4: Confounder analysis using bivariable logistic regression model on clinically significant PK features identified for characterizing hypo/hyper-expression. Confounders: (a) histology, (b) nodal status

(a) histology

Feature	Odds	Log Odds	95% CI	P>|z|
**Median (K^{trans})**	**8.40×10^{-4}**	**-7.0826**	**[-13.836, -0.329]**	**0.040**
90th Percentile (K^{trans})	7.10×10^{-2}	-2.6454	[-5.407, 0.116]	0.060
**Standard Deviation (K^{trans})**	**9.36×10^{-5}**	**-9.2761**	**[-17.926, -0.627]**	**0.036**
**Median (K_{ep})**	**1.33×10^{-1}**	**-2.0144**	**[-3.879, -0.149]**	**0.034**
90th Percentile (K_{ep})	5.25×10^{-1}	-0.6444	[-1.367, 0.078]	0.080

(b) nodal status

Feature	Odds	Log Odds	95% CI	P>|z|
**Median (K^{trans})**	**9.95×10^{-4}**	**-6.9123**	**[-13.237, -0.588]**	**0.032**
90th Percentile (K^{trans})	6.47×10^{-2}	-2.7386	[-5.508, 0.031]	0.053
**Standard Deviation (K^{trans})**	**7.89×10^{-5}**	**-9.4472**	**[-18.329, -0.566]**	**0.037**
**Median (K_{ep})**	**1.43×10^{-1}**	**-1.9430**	**[-3.645, -0.241]**	**0.025**
90th Percentile (K_{ep})	4.92×10^{-1}	-0.7102	[-2.742, 2.395]	0.064
